# Optimization of Natural Deep Eutectic Solvent-Assisted Extraction of Rosmarinic Acid from *Thunbergia laurifolia* Lindl. and Evaluation of Antioxidant Activity

**DOI:** 10.3390/molecules30244795

**Published:** 2025-12-16

**Authors:** Krittima Kriengsaksri, Wisuwat Thongphichai, Tamonwan Uttarawichien, Jasadakorn Khoochonthara, Pasarapa Towiwat, Suchada Sukrong

**Affiliations:** 1 MSc. Program in Research for Enterprise, Faculty of Pharmaceutical Sciences, Chulalongkorn University, Bangkok 10330, Thailand; 2 Center of Excellence in DNA Barcoding of Thai Medicinal Plants, Faculty of Pharmaceutical Sciences, Chulalongkorn University, Bangkok 10330, Thailand; 3 Department of Pharmacognosy and Pharmaceutical Botany, Faculty of Pharmaceutical Sciences, Chulalongkorn University, Bangkok 10330, Thailand; 4 Herb Guardian Co., Ltd., Nonthaburi 11120, Thailand; 5 Institute of Nutrition, Mahidol University, Nakhon Pathom 73170, Thailand; 6 Faculty of Medicine, Vongchavalitkul University, Nakhon Ratchasima 30000, Thailand; 7 Animal Models of Chronic Inflammation-Associated Diseases for Drug Discovery Research Unit, Faculty of Pharmaceutical Sciences, Chulalongkorn University, Bangkok 10330, Thailand; 8 Department of Pharmacology and Physiology, Faculty of Pharmaceutical Sciences, Chulalongkorn University, Bangkok 10330, Thailand; 9 Chulalongkorn School of Integrated Innovation, Chulalongkorn University, Bangkok 10330, Thailand

**Keywords:** *Thunbergia laurifolia*, natural deep eutectic solvent (NaDES), rosmarinic acid, ultrasonic-assisted extraction, keratinocytes, antioxidant activity, chemical stability

## Abstract

*Thunbergia laurifolia* Lindl. is a plant known for its promising biological activity, including antioxidant and anti-inflammatory activities, and a rich source of rosmarinic acid (RA). The extraction of *T. laurifolia* for cosmetic and skincare products using conventional solvents has encountered difficulties, including safety concerns, skin irritation, undesirable odors, and inefficient extraction. In this work, 14 types of natural deep eutectic solvents (NaDESs) with varying compositions and ratios were investigated to compare their efficiency in extracting RA from *T. laurifolia* by HPLC analysis. The NaDES with the highest extraction efficiency was further utilized in ultrasonic-assisted extraction (UAE), and the extraction parameters were optimized using response surface methodology. The optimized RA content and DPPH scavenging activity were predicted by response surfaces methodology to be 7.52 mg/g DW and 37.6 mg TE/g DW, respectively. The optimal extraction condition was achieved using a propylene glycol-lactic acid NaDES (at an 8:1 molar ratio) with 37% (*w*/*w*) H_2_O, a 30 mL/g liquid-to-solid ratio, an 80 °C extraction temperature, and a 32 min extraction time. The optimized extract was proved to suppress ROS in H_2_O_2_-induced keratinocytes. The extract demonstrated robust stability against basic, oxidative, and photolytic stresses, and maintained long-term chemical stability up to 90 days. This study introduces a new green solvent for the effective extraction of *T. laurifolia*, thereby improving the safety and quality of the extracts for skincare and cosmetic products.

## 1. Introduction

The market for skincare products has grown rapidly as global industrial activity has significantly intensified environmental pollution, resulting in a harsher environment that directly impacts cutaneous health [1]. The popularity of cosmetics and skincare products also arises from an increasing focus on self-esteem and the growing prioritization of self-care. Moreover, heightened consumer concern regarding harmful synthetic chemicals in these products [2] has prompted cosmetics and skincare companies to reformulate their products, replacing synthetic compounds with natural or plant-derived alternatives. As increasing marketing demand drives extensive research into the safety and efficacy of natural and green raw materials, synthetic active compounds are being replaced by various plant extracts with numerous biological activities to capture greater customer attention [3,4].

**Figure 1:**
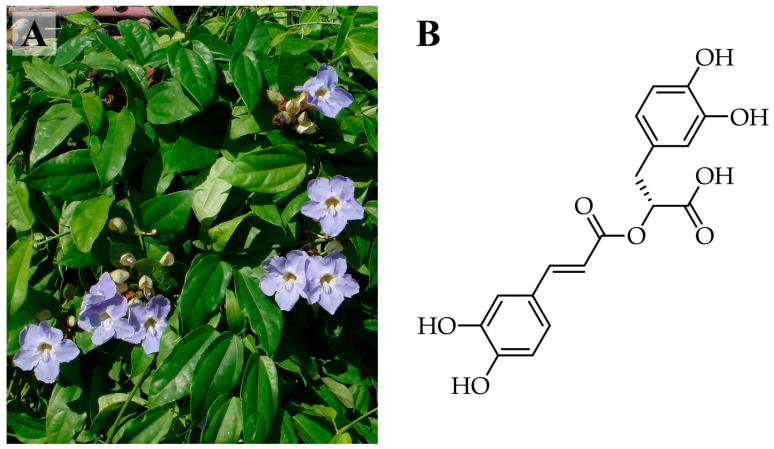
Characteristics of *Thunbergia laurifolia* and its major bioactive constituent. (**A**) The aerial part of *T. laurifolia* showing its flowers and leaves, and (**B**) the chemical structure of rosmarinic acid.

*Thunbergia laurifolia* Lindl. (Figure 1A) is a medicinal plant belonging to the Acanthaceae family. This plant has been cultivated in Southeast Asian countries, including Thailand and Malaysia [5]. *T. laurifolia* leaves are well known for various biological properties, such as antioxidant, detoxifying, and anti-inflammatory activities [6], which are primarily due to their high levels of phenolics and flavonoids. Rosmarinic acid (RA) (Figure 1B) is one of the bioactive phenolics in *T. laurifolia*, which has been reported for its potent antioxidant and anti-inflammatory activities, attributed to its catechol phenolic hydroxyl groups [7]. Consequently, it has been recognized as a reference standard for the quality evaluation and standardization of *T. laurifolia* extracts [8]. These properties make RA and *T. laurifolia* compelling ingredients for skincare products.

Conventional solvents, such as ethyl acetate, acetone, methanol, ethanol, and water, have been used in the extraction of *T. laurifolia*. However, some of these solvents pose toxicity to humans, are environmentally harmful [9], and are not suitable for skincare and cosmetic applications. Although ethanol is considered to have low toxicity, its application is constrained due to its potential to cause skin irritation and dryness, particularly in sensitive skin [10]. Water is also a safe option. However, it is highly polar and only suitable for only a narrow polarity range, leading to a low extraction yield [11]. Aqueous extracts are also prone to microbial growth if not concentrated or stabilized in time. Thus, additional steps to remove these solvents are necessary to avoid toxicity and concentrate the extracts, making the process energy-intensive and time-consuming. To avoid these drawbacks, various alternative solvents were studied, including supercritical fluids, ionic liquids, and natural deep eutectic solvents (NaDESs).

NaDESs are deep eutectic solvents that comprise two or more natural components, such as sugars, choline chloride, organic acids, and glycols. NaDESs exist in liquid form, where the intermolecular interactions between a hydrogen bond acceptor (HBA) and a hydrogen bond donor (HBD) are present [12]. Due to their natural compositions, NaDESs exhibit low to non-toxicity compared to other types of extraction solvents [13]. Consequently, they can be directly incorporated into various topical formulations [14] and implemented as alternative extraction solvents for skincare products. The use of NaDESs instead of conventional solvents eliminates the need for additional purification steps and preserves the natural integrity of the extract [15].

Despite the availability and extensive studies of numerous NaDESs, research focusing on the extraction of phenolic compounds from *T. laurifolia* using these solvents remains scarce [16]. Furthermore, studies on optimizing rosmarinic acid (RA) content and antioxidant activity of NaDES-assisted *T. laurifolia* extracts are limited, and their chemical stability has not yet been investigated. Thus, this study aims to develop ultrasonic-assisted extraction of *T. laurifolia* using various types of NaDESs. The effects of extraction parameters, namely water content, liquid-to-solid ratio, temperature, and time, on RA content and DPPH scavenging activity were further investigated by one-factor experiment and response surface methodology using the Box–Behnken design. Moreover, the chemical stability of an optimized NaDES-assisted extract was evaluated using stress tests and storage tests. Finally, to validate the extract’s potential as a skincare ingredient, its protective effect against oxidative stress was assessed using an intracellular ROS assay in human keratinocytes. This study fulfills the gap in optimizing RA content and antioxidant activity of NaDES-assisted *T. laurifolia* extract, which supports the applications of *T. laurifolia* extract in skincare products.

## 2. Results and Discussion

### 2.1. The Suitable NaDES Combination for Ultrasonic-Assisted Extraction of T. laurifolia Was Determined by Rosmarinic Acid Content and DPPH Scavenging Activity

In this work, we propose the possibility of using NaDES as an alternative solvent for plant extractions, which may be preferred for use as a raw material in cosmetic and skincare products. This approach aims to overcome the limitations of conventional solvents, such as mismatched polarity with target molecules, leading to a low extraction yield. To ensure suitability for these applications, the ingredients of NaDES in this study were meticulously selected based on their safety, aligning with international cosmetic regulations and databases [17,18,19].

**Figure 2:**
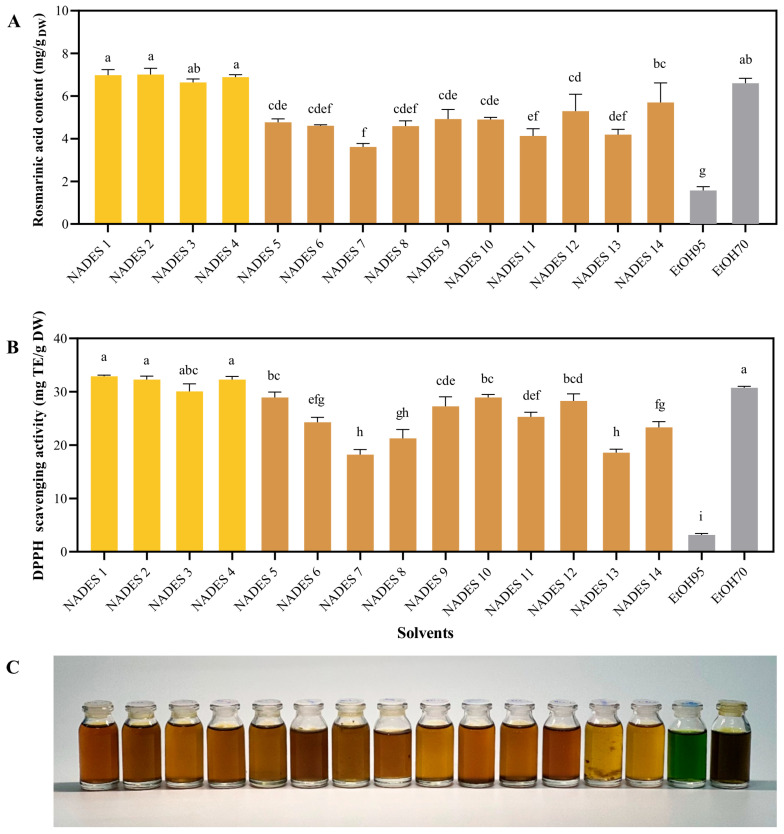
The Extraction performance of fourteen distinct NaDESs and ethanol solutions (95% and 70% *v*/*v*) at a liquid-to-solid ratio of 30 mL/g, 30% *w*/*w* water content, and 50 °C for 15 min. Rosmarinic acid content (**A**), DPPH scavenging activity (**B**), and the physical appearance (**C**) of corresponding extracts obtained using various solvents. Different letters (a, b, c, d, e, f, g, h) designate significant statistical differences (*p* < 0.05). The compositions of each NaDES are listed in Table 1.

To obtain the most suitable NaDES, fourteen NaDES were synthesized and used for UAE of *T. laurifolia* (Table 1). Aqueous ethanol solutions (70% and 95%), which are conventional solvents for plant extraction, were also used for comparison (Figure 2). All extracts were subjected to RA content evaluation using a validated HPLC method. The validation results were shown in Figure S1 and Tables S1–S3. Among the screened solvents, NaDES 1–4, and 70% EtOH extract contained the highest RA content and exhibited the strongest DPPH scavenging activity, compared to the other solvents (Figure 2A). NaDES 1–4 also showed very high DPPH scavenging activity compared with other NaDESs and 70% EtOH (Figure 2B). In contrast, 95% EtOH possessed the lowest in RA content and DPPH scavenging activity. The results suggested that the superior performance of NaDES 1–4 in extracting RA could be attributed to their lactic acid component. Regarding physical appearance, all NaDES extracts appeared as yellow to brown solutions, whereas 95% and 70% ethanolic extracts were green and dark green solutions, respectively (Figure 2C). The green color in ethanolic extracts was attributed to chlorophyll, which is highly soluble in alcohol. This undesirable pigment not only compromises the esthetic appearance of cosmetic products but also pose analytical challenges. Specifically, the strong visible light absorption of chlorophyll and its degradation products can interfere with biological assays [20]. Therefore, additional steps are neccessary for removing these pigments, resulting in increased cost, longer processing time, and reduced extraction yield. These results suggest the applicability of NaDES 1–4 in producing high-quality extracts with an appealing appearance.

**Figure 3:**
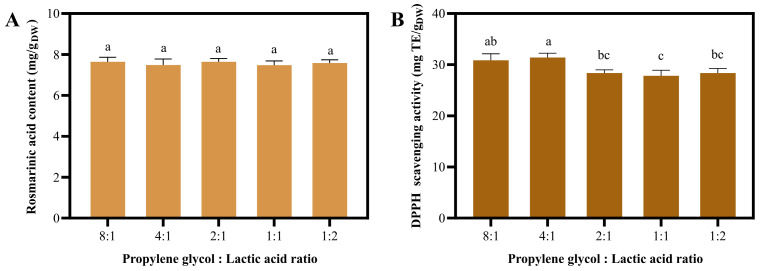
The Impact of the NaDES 4 composition (propylene glycol:lactic acid ratio) on extraction efficiency. Rosmarinic acid content (**A**) and DPPH scavenging activity (**B**) were obtained using various composition ratios. Different letters (a, b, and c) show significant statistical differences (*p* < 0.05).

Although NaDES 1–4 exhibited comparable RA content and antioxidant activity, NaDES 1 was less desirable due to the unpleasant odor of choline chloride. Furthermore, NaDES 2, containing propanediol, is more expensive than NaDES 4, which consists of propylene glycol (Table S9). NaDES 3 contains glycerol, which makes it more viscous than the other NaDESs. Therefore, NaDES 4 was selected to investigate the effect of PG:LA ratios on the extraction properties (Figure 3). The results demonstrated that NaDES 4 with various PG:La ratios exhibited an insignificant difference in RA content (Figure 3A). Regarding antioxidant actvity, increasing the PG:La ratio led to significantly increase in DPPH scavenging activity. The NaDES extract with an 8:1 ratio exhibited the highest activity, although it was not significantly different from 4:1 ratio (Figure 3B). Despite their comparable performance, the high concentration of lactic acid in the 4:1 ratio could compromise the stability of bioactive compounds and limit the extract’s applicability in final products (e.g., due to skin irritation or pH incompatibility). Furthermore, propylene glycol is more cost-effective than lactic acid. Consequently, we selected the NaDES with an 8:1 PG:LA ratio for the subsequent one-factor experiment.

### 2.2. Optimal Extraction Parameters Were Successively Obtained from One-Factor Experiments

**Figure 4:**
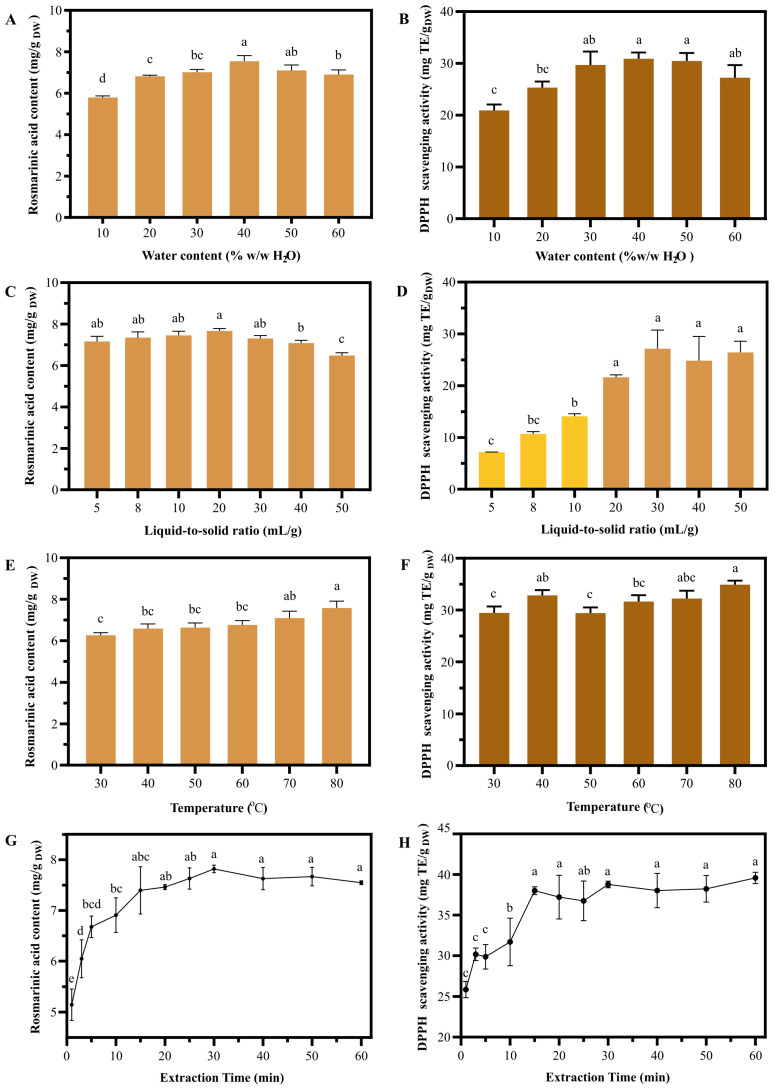
The impact of extraction parameters on *T. laurifolia* extract performance. Rosmarinic acid content and DPPH scavenging activity of NaDES extracts were obtained under varying conditions: water content (**A**,**B**), liquid-to-solid ratio (**C**,**D**), temperature (**E**,**F**), and time (**G**,**H**). Different letters (a, b, and c) indicate significant statistical differences (*p* < 0.05).

To obtain optimal conditions for NaDES-assisted UAE of *T. laurifolia*, the chosen NaDES with selected PG:La ratio was preliminary subjected to one-factor experiments. The variation in water content, liquid-to-solid ratio, temperature, and extraction time led to the results shown in Figure 4. NaDES with 40% H_2_O content showed the highest RA content of 7.55 ± 0.22 mg/g DW, while 10% H_2_O NaDES demonstrated the lowest RA content of 5.79 ± 0.06 mg/g DW (Figure 4A). The maximized DPPH scavenging activity of 30.89 ± 1.21 mg Trolox equivalent (TE)/g DW was also obtained from 40% and 50% H_2_O NaDES, while 10% H_2_O NaDES exhibited the lowest antioxidant activity of 20.91 ± 1.15 mg TE/g DW (Figure 4B). The results indicated that the extraction efficiency of NaDES was reduced when the water content deviated from its optimal level. Insufficient water content resulted in high viscosity, hindering mass transfer between the plant matrix and the solvent [21]. Conversely, excessive water content increased the solvent polarity, potentially leading to a mismatch with the active constituents in the plant material [22]. For the liquid-to-solid (LSR) experiments, the highest RA content of 7.67 ± 0.09 mg/g DW was obtained when 20:1 LSR was used, while 50:1 LSR showed the lowest RA content of 7.17 ± 0.20 mg/g DW (Figure 4C). The 5:1 LSR showed the lowest DPPH scavenging activity of 7.16 ± 0.04 mg TE/g DW, and the activity sharply rose as the LSR increased. At 20:1 LSR, the extract revealed maximized antioxidant activity of 21.64 ± 0.43 mg TE/g DW. However, increasing LSR from 20:1 to 50:1 did not significantly increase antioxidant activity (Figure 4D). The results indicate that a higher LSR could facilitate mass transfer between the plant matrix and the solvent, leading to significant effect RA content and antioxidant activity.

Different extraction temperatures also affected the RA content and antioxidant activity of the extract. The lowest RA content and antioxidant activity (6.26 ± 0.10 mg/g DW and 29.46 ± 1.22 mg TE/g DW) were obtained at 30 °C, and were increased as the temperature rose up to 70 °C. However, the change from 70 °C to 80 °C did not significantly increase the RA content and DPPH scavenging activity (Figure 4E,F). The relationship between RA content, DPPH scavenging activity, and extraction temperature observed in this study aligns with a previous study by Rojsanga et al. on the optimal infusion conditions of *T. laurifolia* [23], suggesting that the extraction temperature has crucial impact on RA content. Since the results obtained at 70 °C and 80 °C were not significantly different, 70 °C was selected for the subsequent extraction optimization to maximize energy efficiency. The extraction time experiment revealed an increasing trend in RA content and antioxidant activity from 1 to 30 min, reaching a maximum RA content of 7.19 ± 0.06 mg/g DW and DPPH scavenging activity of 38.78 ± 0.40 mg TE/g DW. Further increase in extraction time did not significantly alter RA content and antioxidant activity (Figure 4G,H), indicating that the extract was saturated after 30 min. According to the results, the optimized extraction conditions from one-factor experiments, including 40% water content, 20 mL/g liquid-to-solid ratio, 70 °C extraction temperature, and 30 min extraction time, were further assigned as the middle levels in the Box–Behnken design.

### 2.3. The Optimized Extraction Conditions Were Achieved by Response Surface Methodology with Box–Behnken Design

To investigate the relationship between extraction parameters and determine the optimized conditions for RA content, each parameter was varied at three levels (Table 2). The optimized conditions from one-factor experiments were set as the middle level (level 0). Twenty-seven extraction experiments were constructed using a Box–Behnken statistical experimental design (Table S4). The acquired experimental data were fitted to a second-order polynomial equation to obtain the response surface curve for RA content, as shown in Equation (1).

**Figure 5:**
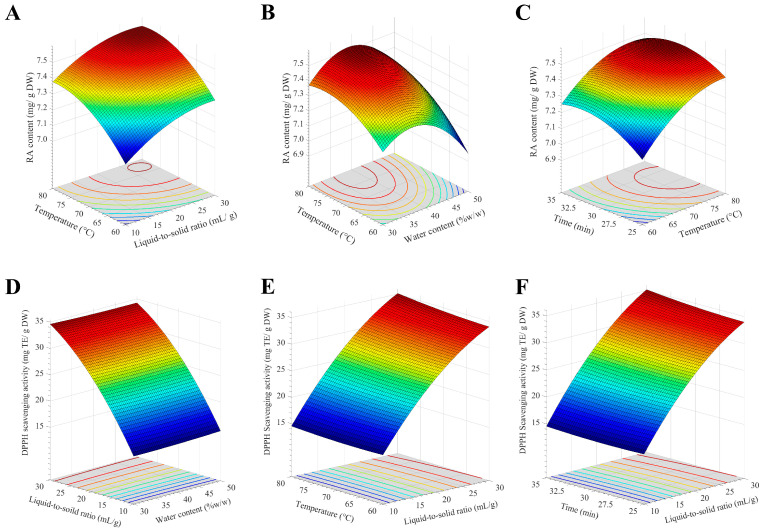
The response surface between extraction parameters, rosmarinic acid (RA) content, and antioxidant activity of *T. laurifolia* extract. (**A**) The effect of temperature and liquid-to-solid ratio (LSR) on RA content. (**B**) The effect of temperature and water content on RA content. (**C**) The effect of temperature and extraction time on RA content. (**D**) The effect of LSR and water content on DPPH scavenging activity. (**E**) The effect of temperature and LSR on DPPH scavenging activity. (**F**) The effect of extraction time and LSR on DPPH scavenging activity.

(1)        RA content (mg/g DW) = − 5.0306 + 0.15080 **W** + 5.8465 × 10^−2^ **L** + 0.12968 **T** + 0.25368 **M**

                       − 5.0639 × 10^−4^ **W** × **L** + 3.9121 × 10^−4^ **W** × **T** − 9.3343 × 10^−4^ **W** × **M**

                       − 1.2810 × 10^−4^ **L** × **T** + 1.2070 × 10^−4^ **L** × **M** − 5.1194 × 10^−4^ **T** × **M**

                − 1.8449 × 10^−3^ **W**^2^ − 5.8046 × 10^−4^ **L**^2^ − 8.1489 × 10^−4^ **T**^2^

− 2.8930 × 10^−3^ **M**^2^

where **W** is the water content, **L** is the liquid-to-solid ratio, **T** is the extraction temperature, and **M** is the extraction time. The statistical analysis of this model for RA content is shown in Tables S5 and S6. The results indicate good correlation between the data by a high F-value, very low *p*-value, and high *p*-value for lack of fit parameter. The R^2^ of the models was in good agreement between the adjusted and predicted values (the difference is less than 0.2), indicating the relevance between the experimental and predicted values. It was found that RA content was influenced mostly by extraction time, followed by water content, extraction temperature, and liquid-to-solid ratio. When water content and extraction time were kept constant, increasing the temperature to 75–80 °C and the liquid-to-solid ratio to 25–30 mL/g led to an increase in RA content to approximately 7.52 mg/g DW (Figure 5A). Regarding the correlation between the temperature and water content (Figure 5B), the RA content was optimized when water content reached 35–40 mL/g. The response surface of extraction time and temperature revealed that the RA content was at an optimum level when the extraction time was between 28 and 32.5 min (Figure 5C). From the response surfaces, the optimized RA content up to 7.52 mg/g DW was obtained when 37% *w*/*w* water content, 29 mL/g liquid-to-solid ratio, 76 °C extraction temperature, and 32 min extraction time were used.

The response surface between extraction parameters and antioxidant activity of the extracts was also investigated and fitted into a second-order polynomial Equation (2).

(2)     DPPH Scavenging activity = 33.3956 + 0.19009 **W** + 1.3686 **L** − 0.26616 **T** − 1.8050 **M**

          (mg TE/g DW)         − 1.9003 × 10^−3^ **W** × **L** − 2.2598 × 10^−3^ **W** × **T** − 2.4652 × 10^−3^ **W** × **M**

                        + 2.1571 × 10^−3^ **L** × **T** + 1.7633 × 10^−2^ **L** × **M** + 1.1642 × 10^−3^ **T** × **M**

                + 1.0129 × 10^−3^ **W**^2 −^ 2.4110 × 10^−2^ **L**^2^ + 2.2541 × 10^−3^ **T**^2^

+ 2.5349 × 10^−2^ **M**^2^

The statistical analysis of this model for DPPH scavenging activity is shown in Tables S7 and S8. The results indicate good correlation between the data with acceptable statistical parameters. The R^2^ of the models was in good agreement between the adjusted and predicted values, indicating the relevance between the experimental and predicted values. The response surface (Figure 5D) exhibited a steep sloop along the LSR axis, indicating that DPPH scavenging activity of *T. laurifolia* extract was primarily influenced by the LSR, while water content exerted a minimal effect. The optimized DPPH scavenging activity reached 35 mg TE/g DW when the LSR and temperature were increased to 30 mL/g and 80 °C, respectively (Figure 5E). DPPH scavenging activity reached its maximum as the extraction time increased to 35 min (Figure 5F). From the response surfaces, the optimized DPPH scavenging activity was 37.6 mg TE/g DW. Based on the simultaneous optimization of RA content and DPPH scavenging activity, the optimal conditions were identified as 37% water content, 30 mL/g liquid-to-solid ratio, 80 °C extraction temperature, and 32 min extraction time. Although the optimized condition requires elevated temperatures, the remarkably short extraction time when compared to conventional extraction methods, suggests a favorable process efficiency. Therefore, *T. laurifolia* leaves were then extracted with optimized conditions and further evaluated for in vitro antioxidant activity.

### 2.4. NaDES-Assisted T. laurifolia Extract Suppresses H_2_O_2_-Induced Intracellular ROS Production

**Figure 6:**
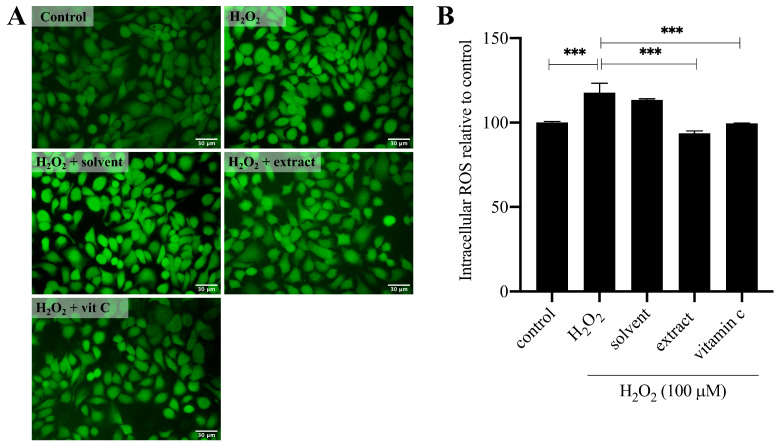
Protective effect of the plant extract against H_2_O_2_-induced intracellular ROS production in keratinocytes. Cells were pre-treated for 24 h with the solvent (0.8% *v*/*v*), plant extract (0.8% *v*/*v*), or vitamin C (400 µM) before being challenged with H_2_O_2_ (100 µM) for 30 min. Intracellular ROS levels were then measured using the H_2_DCFDA probe. (**A**) Representative fluorescence microscopy images showing intracellular ROS as green fluorescence. Scale bar = 30 µm, magnification 200×. (**B**) Quantification of the relative fluorescence intensity from the assay. Data are presented as the mean ± SD of three independent experiments (*n* = 3). *** *p* < 0.001 indicates a significant difference.

To investigate on the potential of *T. laurifolia* NaDES extract as an active ingredient for skin protection, in vitro antioxidant activity on H_2_O_2_-induced human keratinocyte cells was selected as a model. Prior to evaluating any protective effects, the non-toxic concentration of *T. laurifolia* extract was determined using the MTT assay to assess keratinocyte viability (Figure S2). After a 24 h treatment, the non-toxic concentrations were determined to be 100 µM for H_2_O_2_ (93.05 ± 7.41% viability) (Figure S2A), 0.8% *v*/*v* for the NaDES (97.68 ± 1.54%) (Figure S2B), 0.8% *v*/*v* for the *T. laurifolia* NaDEs extract (96.77 ± 2.80%) (Figure S2C), and 400 µM for vitamin C (91.80 ± 2.02%) (Figure S2D), relative to the untreated control group. These concentrations were then used for subsequent intracellular ROS determination experiments using the H_2_DCFDA fluorescent probe. The cells were pre-treated for 24 h with test compounds before being challenged with H_2_O_2_ for 30 min (Figure 6). Fluorescence microscopy provided initial qualitative evidence of the extract’s protective effect. As shown in Figure 6A, cells exposed to H_2_O_2_ alone exhibited an intense green fluorescence, indicative of high intracellular ROS levels. This intense signal was visibly diminished in cells that were pre-treated with either the plant extract or the positive control, vitamin C. These visual observations were confirmed by quantitative analysis. The results showed that H_2_O_2_ treatment significantly increased ROS levels to 117.60 ± 5.57% compared to the untreated control (*p* < 0.05). However, pre-treatment with the plant extract (0.8% *v*/*v*) markedly counteracted this effect, significantly suppressing ROS generation to 93.64 ± 1.39%. This potent protective effect was statistically comparable to that of vitamin C (400 µM), which reduced ROS to 99.60 ± 0.02%, while the solvent vehicle (0.8% *v*/*v*) showed no significant inhibitory activity, compared to the H_2_O_2_-induced group (Figure 6B). The results demonstrated that the NaDES-assisted *T. laurifolia* extract possessed potent intracellular ROS scavenging activity comparable to vitamin C. Since the NaDES itself exhibited negligible antioxidant activity, the observed effect is attributed to the chemical constituents of *T. laurifolia*, including rosmarinic acid. Since ROS is involved in inflammation, skin damage caused by pollution, and aging [24,25,26], The biological activity of *T. laurifolia* NaDES extract could alleviate these skin problems.

### 2.5. NaDES-Assisted T. laurifolia Extract Was Stable Under Stress and Various Storage Conditions

To evaluate the chemical stability of the NaDES-assisted *T. laurifolia* extract, stress and storage tests were performed. The results demonstrate that RA content in NaDES extract remained stable when under basic stress (Table 3), contrasting with previous studies that reported RA instability under alkaline conditions [27,28]. This enhanced chemical stability is potentially attributed to lactic acid in NaDES, which may help neutralize excess alkalinity. Additionally, the extract exhibited resistance to oxidative stress, consistent with the findings of Woottisin et al. [27]. However, the extract is chemically unstable toward acidic stress, as an addition of concentrated acid led to the decomposition of around 20 percent RA content (Table 3). This result agreed with previous studies [27,29], indicating the instability of RA in extreme acidic conditions. The photolytic stress test showed a slight decrease in RA content. Even if the result is insignificant, long-term storage under the light should be avoided to preserve the quality of an extract [30].

**Figure 7:**
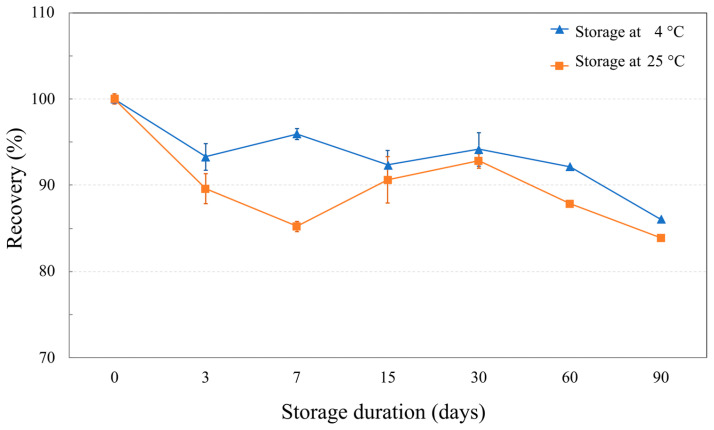
The line graphs show rosmarinic acid recovery (%) in NaDES-assisted *T. laurifolia* extract at various storage durations.

Storage test also disclosed long-term stability of NaDES-assisted extract (Figure 7). At 4 °C, rosmarinic acid was significantly decreased to 93.31 ± 1.54% on day 3. The RA recovery then remained relatively stable until day 60, after which further decreases were observed to 86.06 ± 0.17% on day 90. At ambient storage temperature (25 °C), the results showed that the RA recovery trend was the same as at 4 °C, but overall RA recovery was lower. The slow degradation rate of RA in the *T. laurifolia* NaDES extract highlights its stability over a long-term storage. Given that an extract stored at ambient temperature experience greater RA loss compare to lower temperature, storage conditions significantly impact the quality of the extract.

## 3. Materials and Methods

### 3.1. Chemicals

Lactic acid (La; cat. no. CF1201), citric acid (Cit; cat. no. CA0310), glucose (Glu; cat. no. FS0402), sorbitol (Sorb; cat. no. FS1905), and propylene glycol (PG; cat. no. CA1612) were purchased from Chemipan (Bangkok, Thailand). Choline chloride (ChCl; cat. no. 02800) was acquired from Loba Chemie (Mumbai, India). Glycerol (Gly) was garnered from Wittayasom (Bangkok, Thailand). Zemea^®^ propanediol (PD) was obtained from CovationBio PDO (Loudon, TN, USA). Ethanol was purchased from T.S. Interlab LP (Bangkok, Thailand). 2,2-diphenyl-1-picrylhydrazyl (DPPH; cat. no. D9132) and ascorbic acid (VitC; cat. no. A92902) were obtained from Sigma-Aldrich (St. Louis, MO, USA). Trolox (cat. no. H0726) was acquired from TCI (Tokyo, Japan). HPLC grade methanol (cat. no. AH230-4A) was purchased from Honeywell (Charlotte, NC, USA). Acetic acid (cat. no. 100063) and DMSO (cat. no. 102952) were garnered from Merck (Darmstadt, Germany). Standard rosmarinic acid (RA; cat. no. 536954) was obtained from Sigma-Aldrich (St. Louis, MO, USA). Ultrapure water was produced from a Barnstead ™ MicroPure ™ Water Purification System (Thermo Fisher Scientific, Waltham, MA, USA). Human keratinocytes (cat. no. 300493) were received from CLS Cell Lines Service GmbH (Eppelheim, Germany). Dulbecco’s modified Eagle’s medium with high glucose (DMEM; cat. no. D5796) and 2′,7′-dichlorodihydrofluorescein diacetate (H_2_DCFDA; cat. no. 35845) were purchased from Sigma-Aldrich (St. Louis, MO, USA). MTT solution (cat. no. M6494), penicillin-streptomycin (cat. no. 1IVG7-15140-122), GlutaMAX reagent (cat. no. 1IVG7-35050-061), and fetal bovine serum (FBS; cat. no. F0804) were acquired from Thermo Fisher Scientific (Waltham, MA, USA). Dulbecco’s phosphate-buffered saline (DPBS; cat. no. 21-030-CM) was obtained from Corning^®^ (Glendale, AZ, USA).

### 3.2. Plant Materials

*Thunbergia laurifolia* leaves were purchased from a local market in Nan Province, Thailand. The sample was identified by Associate Professor Thatree Phadungcharoen, a taxonomist at the Faculty of Pharmaceutical Sciences, Chulalongkorn University. The fresh leaves were cleaned and put in an air-dry oven at 50 °C for 24 h. The dried leaves were further milled into a fine powder and stored in sealed containers under desiccated and light-protected conditions.

### 3.3. Preparing and Screening of NaDES

NaDES were prepared by using ultrasound-assisted synthesis [31]. Briefly, pre-weighed hydrogen bond donors (HBDs) and acceptors (HBAs) at specified molar ratios and deionized water were mixed, followed by sonication in GT SONIC D-series ultrasonic cleaner (GuangDong GT Ultrasonic Co., Ltd., Shenzhen, China; 40 kHz, 500 W) for typically 15–30 min until homogeneous solutions were formed. The solutions were stored in sealed containers at ambient temperature until used.

For screening optimal NaDES for *T. laurifolia* extraction, various acid- and sugar-based NaDES were prepared, maintaining a 1:1 molar ratio of HBA to HBD and containing 30% *w*/*w* water. Ethanol solutions at concentrations of 70% and 95% (*v*/*v*) were used to represent conventional solvents. The list of all NaDES in this study is presented in Table 1. Then, 1.00 g of *T. laurifolia* powder was combined with 30 mL of each NaDES, aqueous ethanol solution, or deionized water in an amber glass bottle. Then the mixtures were sonicated in an ultrasonic bath at 50 °C for 15 min of extraction time. The mixtures were filtered through a tea bag filter. After the extraction, the extract solution was analyzed for RA content and antioxidant activity. The extraction solvent, which shows the highest RA content and antioxidant activity, was further investigated on the effect of molar ratios. The HBA:HBD molar ratios of the selected NaDES were varied to 8:1, 4:1, 2:1, 1:1, and 1:2. The NaDES provides the highest RA content, and antioxidant activity was chosen for one-factor experiments.

### 3.4. Determination of Rosmarinic Acid Content Using HPLC

Rosmarinic acid (RA) content in the *T. laurifolia* extracts, obtained using various NaDESs and extraction conditions, was determined by HPLC using an Agilent 1290 Infinity II UHPLC system equipped with a Zorbax Eclipse Plus C-18 reversed-phase column (250 mm × 4.6 mm, 5 µm; Agilent, Santa Clara, CA, USA) and an appropriate C-18 guard column. The mobile phases consisted of 0.5% acetic acid (A) and MeOH (B). All mobile phases were filtered through a 0.22 μm nylon filter (Vertical, Bangkok, Thailand, cat. no. 0235–0101) before use. Separation of RA was achieved by the gradient elution as follows: 0–15 min, 25–65% B; 15–20 min, 65–96% B. The flow rate was 1.0 mL/min, and the injection volume was 10 μL. The column temperature was maintained at ambient temperature (25 °C), and a diode array detector was used to monitor the UV absorption of RA at 325 nm [27]. A standard stock solution of RA was prepared by dissolving 1 mg of RA in 1 mL of MeOH to obtain a concentration of 1 mg/mL. Working solutions of RA with concentrations of 10, 25, 50, 100, 250, and 500 were prepared by dilution of the stock solution. A calibration curve of RA was constructed by plotting the peak area of the RA standard against its corresponding concentration. The RA content in the *T. laurifolia* extracts was calculated by their RA peak area with a calibration curve and expressed as milligrams of rosmarinic acid per gram of dry weight of plant material (mg/g DW). All analyses were performed in triplicate.

### 3.5. Evaluation of Antioxidant Activity of T. laurifolia Extracts

The antioxidant activity of the *T. laurifolia* extracts, obtained from various extraction conditions, was evaluated using the 2,2-Diphenyl-1-picrylhydrazyl (DPPH) radical scavenging assay as previously described, with slight modifications [32]. Briefly, a 150 µM DPPH solution was prepared by dissolving DPPH in MeOH. Each *T. laurifolia* extract was prepared by ten-fold dilution in MeOH, then 20 µL of each diluted extract was mixed with 180 µL of the DPPH solution in a 96-well plate. The mixtures were incubated in the dark at room temperature for 30 min, then the absorbance was measured at 515 nm using CLARIOstar^®^ Plus microplate reader (BMG Labtech, Ortenberg, Germany). MeOH was used as a blank, while Trolox and VitC were used as positive controls. The DPPH radical scavenging activity was calculated using the following formula:

DPPH scavenging activity (%) = [(A_control_ − A_sample_)/A_control_] × 100 (3)

where A_control_ is the absorbance of the DPPH solution without the extract, and A_sample_ is the absorbance of the DPPH solution with the extract or positive controls. All measurements were performed in triplicate.

### 3.6. One Factor Experiments

To investigate the influence of individual extraction parameters on the yield of RA extracted using the selected NaDES, one-factor experiments were conducted [33]. In each experiment, one parameter was varied while the others were kept constant. The factors investigated in this study were water content in NaDES (10–60% *w*/*w*), liquid to solid ratio (LSR, 5–50 mL NaDES/g of *T. laurifolia* powder), extraction temperature (30–80 °C), and time (3–60 min), respectively. After each experiment, the RA content was determined using HPLC as previously described. The level of the factor that yielded the extracts with the highest RA content was considered optimal.

### 3.7. Multi-Factor Experimental Designs and Response Surface Methodology (RSM)

Box–Behnken design (BBD) was further employed to find the optimized condition for the UAE process [34]. Each parameter was divided into three levels: low (−1), middle (0), and high (+1). The middle levels of each parameter were adopted from optimal conditions obtained from one-factor experiments, whereas low- and high-levels were the boundary values. Twenty-seven experiments with three center points were conducted, and the results were applied to regression analysis using a second-order polynomial model [34] (Equation (4)).

(4) $$Y=b_{0}+\sum_{i=1}^{k}b_{i}X_{i}+\sum_{i=1}^{k}b_{ii}X_{i}^{2}+\sum_{i=1}^{k-1}\sum_{j=i+1}^{k}b_{ij}X_{i}X_{j}$$

where *Y* is the response variable (RA content and %DPPH scavenging activity); *b*_0_, *b_i_*, *b_ii_*, and *b_ij_* are the regression coefficients for intercept, linear, quadratic, and interaction terms, respectively; *k* is the number of independent variables (*k* = 4); *Xi* and *Xj* represent the independent variables (*i* ≠ *j*).

Statistical models were constructed using MATLAB R2024a software (MathWorks, Natick, MA, USA). In addition, response surface plots were generated and visualized to indicate the relationship between each experimental factor and suggest the best conditions for NaDES-assisted extraction of *T. laurifolia*.

### 3.8. Reactive Oxygen Species Scavenging Assay on H_2_O_2_-Induced Keratinocytes

#### 3.8.1. Cell Culture

Human keratinocytes (Cat. No. 300493; CLS Cell Lines Services, Eppelheim, Germany) were cultured in high-glucose (4.5 g/L) Dulbecco’s Modified Eagle’s Medium (DMEM). The growth medium was supplemented with 10% (*v*/*v*) fetal bovine serum (FBS), 2 mM L-glutamine, and 100 U/mL penicillin-streptomycin. Cultures were maintained at 37 °C in a humidified atmosphere containing 5% CO_2_.

#### 3.8.2. Cell Viability Assay

The viability of keratinocytes was assessed using the 3-(4,5-dimethylthiazol-2-yl)-2,5-diphenyltetrazolium bromide (MTT) assay to determine the non-toxic concentration of the test compounds [35]. Cells were seeded in 96-well plates at a density of 1.0 × 10^4^ cells/well and incubated overnight. The following day, the culture medium was replaced with medium containing varying concentrations of H_2_O_2_, solvent (vehicle control), the plant extract, or vitamin C, and the cells were incubated for 24 h. Following the treatment period, MTT solution was added to each well to a final concentration of 0.5 mg/mL and incubated for an additional 2 h. The resulting formazan crystals were then solubilized with dimethyl sulfoxide (DMSO), and the absorbance was quantified at 570 nm using a CALIOSTAR microplate reader (BMG Labtech, Ortenberg, Germany).

#### 3.8.3. Measurement of Intracellular ROS

Intracellular ROS levels were quantified using the fluorescent probe 2′,7′-dichlorodihydrofluorescein diacetate (H_2_DCFDA). Keratinocytes were seeded in 96-well plates at a density of 1.0 × 10^4^ cells/well and incubated overnight. The cells were then pre-treated for 24 h with the solvent (vehicle control), the plant extract, or vitamin C (positive control, 400 µM) prior to inducing oxidative stress with H_2_O_2_ for 30 min. After treatment, cells were washed with phosphate-buffered saline (PBS) and incubated with 10 μM H_2_DCFDA for 30 min in the dark. Following a final PBS wash to remove the excess probe, the fluorescence intensity of the oxidized product, dichlorofluorescein (DCF), was measured using a CALIOSTAR microplate reader with excitation and emission wavelengths of 485 nm and 535 nm, respectively. For visualization, fluorescence images were captured from randomly selected fields using an Olympus IX51 microscope (Olympus Corporation, Centervalley, PA, USA). ROS levels were quantified relative to the untreated control cells.

### 3.9. Chemical Stability Study of NaDES-Assisted T. laurifolia Extract

To investigate the chemical stability, *T. laurifolia* extract was exposed to various types of stress, including acidic, basic, oxidative, and photolytic conditions [27]. The acidic, basic, and oxidative stress tests were conducted by adding 50 µL of 37% hydrochloric acid, 5 N sodium hydroxide solution, and 30% (*w*/*w*) hydrogen peroxide solution to 1 mL of NaDES-assisted *T. laurifolia* extract. These conditions corresponded to final concentrations of 0.57 M HCl, 0.24 M NaOH, and 0.47 M H_2_O_2_, respectively. Each mixture was placed into a 60 °C water bath for 60 min. A mixture of 1 mL NaDES-assisted extract with 50 µL of deionized water was used as a control. The photolytic test was executed by placing the extract under a fluorescent lamp (4000 Lux) for 3 days. The experiment was performed in triplicate, and then the extracts were subjected to evaluate the recovery of rosmarinic acid.

The optimized NaDES-assisted extract was evaluated for its stability upon long-term storage. The aliquots from *T. laurifolia* extract were divided into two groups. The first group was put in a refrigerator at 4 °C. The second group was left at ambient temperature (25 °C). All groups were kept in the dark and were sampled at 0, 3, 7, 15, 30, 60, and 90 days to evaluate RA recovery.

### 3.10. Statistical Analysis

All experiments were performed in triplicate and reported as mean ± S.D. One-way ANOVA with Tukey’s or Dunnett’s test was performed in GraphPad Prism software (9.0.0) for comparison of the results.

## 4. Conclusions

This study suggests the potential NaDES for rosmarinic acid-rich *T. laurifolia* extract possessing high antioxidant activity. The optimized parameters for UAE were successfully determined using one-factor experiments, and their effects on RA content and DPPH scavenging activity were disclosed using response surface methodology. NaDES-assisted extract of *T. laurifolia* leaves was also proved to reduce reactive oxygen species on H_2_O_2_-induced human keratinocytes, which play an important role in inflammation, skin aging, and skin damage from pollution. In addition, NaDES-assisted extract revealed desirable chemical stability against basic, oxidative, and photolytic stress. The extract efficiently preserves RA content more than 85 percent after 90 days of storage. This achievement can be used as support for the application NaDES-assisted extract of *T. laurifolia* and other medicinal plants as raw materials in cosmetic and skincare products. However, further investigations into the physicochemical properties of these NaDESs are recommended to understand the solvent mechanisms facilitating bioactive compound extraction. Moreover, comprehensive safety assessments are essential to establish the *T. laurifolia* NaDES extract as a viable ingredient for skincare products.

## Figures and Tables

**Table 1:** Composition of NaDES and conventional solvents used in this study.

No.	Name of NaDES	Component 1	Component 2
1	ChCl:La	Choline chloride	Lactic acid
2	PD:La	Propanediol	Lactic acid
3	Gly:La	Glycerol	Lactic acid
4	PG:La	Propylene glycol	Lactic acid
5	ChCl:Glu	Choline chloride	Glucose
6	PD:Glu	Propanediol	Glucose
7	Gly:Glu	Glycerol	Glucose
8	PG:Glu	Propylene glycol	Glucose
9	ChCl:Sorb	Choline chloride	Sorbitol
10	PD:Sorb	Propanediol	Sorbitol
11	Gly:Sorb	Glycerol	Sorbitol
12	PG:Sorb	Propylene glycol	Sorbitol
13	Cit:Sorb	Citric acid	Sorbitol
14	La:Sorb	Lactic acid	Sorbitol
15	EtOH95	95% (*v*/*v*) Aqueous ethanol	
16	EtOH70	70% (*v*/*v*) Aqueous ethanol	

Each NaDES contain a 1:1 molar ratio of component 1 and component 2 and 30% (*w*/*w*) water content. EtOH70 and EtOH95 were used as representative conventional extraction solvents.

**Table 2:** The levels of independent variables and corresponding values employed in the Box–Behnken design for response surface methodology.

Factors	Units	Symbols		Levels	
			−1	0	1
Water content	% *w*/*w*	**W**	30	40	50
Liquid-to-solid ratio	mL/g	**L**	10	20	30
Extraction temperature	°C	**T**	60	70	80
Extraction time	min	**M**	25	30	35

**Table 3:** Stress tests and %recovery of rosmarinic acid in NaDES-assisted *T. laurifolia* extract.

Stress Type	Reagent	Condition	%Recovery
Control	-	-	100.00 ± 0.39
Acidic	37% HCl	60 °C, 60 min	81.77 ± 0.44 ***
Basic	5N NaOH	60 °C, 60 min	100.19 ± 3.63
Oxidative	30% H_2_O_2_	60 °C, 60 min	101.61 ± 3.81
Photolytic	-	Fluorescent lamp 4000 Lux, ambient (25 °C), 72 h	95.72 ± 2.04

*** *p* < 0.001 indicates a significant difference.

## Data Availability

The original contributions presented in this study are included in the article/Supplementary Materials. Further inquiries can be directed to the corresponding author.

## Supplementary Materials

The following supporting information can be downloaded at: https://www.mdpi.com/article/10.3390/molecules30244795/s1, Figure S1: HPLC quantitative analysis of rosmarinic acid (RA) in *T. laurifolia* extract; Figure S2: Effect of a 24 h treatment with test compounds on the viability of keratinocytes; Table S1: Properties of calibration curves, calculated LOD, LOQ, and range of quantitation of rosmarinic acid; Table S2: Intra-day and inter-day precision of rosmarinic acid; Table S3: Accuracy test result of rosmarinic acid; Table S4: Experimental plan used for the optimization by Box–Behnken Design (BBD); Table S5: ANOVA of quadratic model from BBD for rosmarinic acid content; Table S6: Estimated Coefficients and statistics of each coefficient for quadratic model from BBD for rosmarinic acid content; Table S7: ANOVA of quadratic model from BBD for DPPH scavenging activity; Table S8: Estimated Coefficients and statistics of each coefficient for quadratic model from BBD for DPPH scavenging activity; Table S9: The price per unit of chemicals used as an ingredient for NaDES in this study.

## Abbreviations

The following abbreviations are used in this manuscript:

Cit	Citric acid
ChCl	Choline chloride
DMEM	Dulbecco’s modified Eagle’s medium
DPBS	Dulbecco’s phosphate-buffered saline
DPPH	2,2-diphenyl-1-picrylhydrazyl
FBS	Fetal bovine serum
Glu	Glucose
Gly	Glycerol
H_2_DCFDA	2′,7′-dichlorodihydrofluorescein diacetate
La	Lactic acid
NaDES	natural deep eutectic solvent
PD	Propandiol
PG	Propylene glycol
RA	Rosmarinic acid
Sorb	Sorbitol
VitC	Ascorbic acid
