# Supplementary material for: Optimization of Natural Deep Eutectic Solvent-Assisted Extraction of Rosmarinic Acid from Thunbergia laurifolia Lindl. and Evaluation of Antioxidant Activity
Source: Molecules. 2025 Dec 16;30(24):4795. doi: 10.3390/molecules30244795 (PMC12736046; doi:10.3390/molecules30244795)
Supplement: Supplementary file 1 [file molecules-30-04795-s001.zip › molecules-3990075-supplementary.pdf]

Supporting Materials

Optimization of Natural Deep Eutectic Solvent-Assisted Extraction of Rosmarinic Acid from *Thunbergia Laurifolia* Lindl. and Evaluation of Antioxidant Activity

Krittima Kriengsaksri [1,2,3,4], Wisuwat Thongphichai [2,3,5], Tamonwan Uttarawichien [2,3,6], Jasadakorn Khoochonthara [2,3], Pasarapa Towiwat [7,8] and Suchada Sukrong [2,3,9,*]

[1] MSc. Program in Research for Enterprise, Faculty of Pharmaceutical Sciences, Chulalongkorn University, Bangkok 10330, Thailand

[2] Center of Excellence in DNA Barcoding of Thai Medicinal Plants, Faculty of Pharmaceutical Sciences, Chulalongkorn University, Bangkok 10330, Thailand

[3] Department of Pharmacognosy and Pharmaceutical Botany, Faculty of Pharmaceutical Sciences, Chulalongkorn University, Bangkok 10330, Thailand

[4] Herb Guardian Co., Ltd., Nonthaburi 11120, Thailand

[5] Institute of Nutrition, Mahidol University, Nakhon Pathom 73170, Thailand

[6] Faculty of Medicine, Vongchavalitkul University, Nakhon Ratchasima 30000, Thailand

[7] Animal Models of Chronic Inflammation-Associated Diseases for Drug Discovery Research Unit, Faculty of Pharmaceutical Sciences, Chulalongkorn University, Bangkok 10330, Thailand

[8] Department of Pharmacology and Physiology, Faculty of Pharmaceutical Sciences, Chulalongkorn University, Bangkok 10330, Thailand

[9] Chulalongkorn School of Integrated Innovation, Chulalongkorn University, Bangkok 10330, Thailand

* Correspondence: suchada.su@chula.ac.th

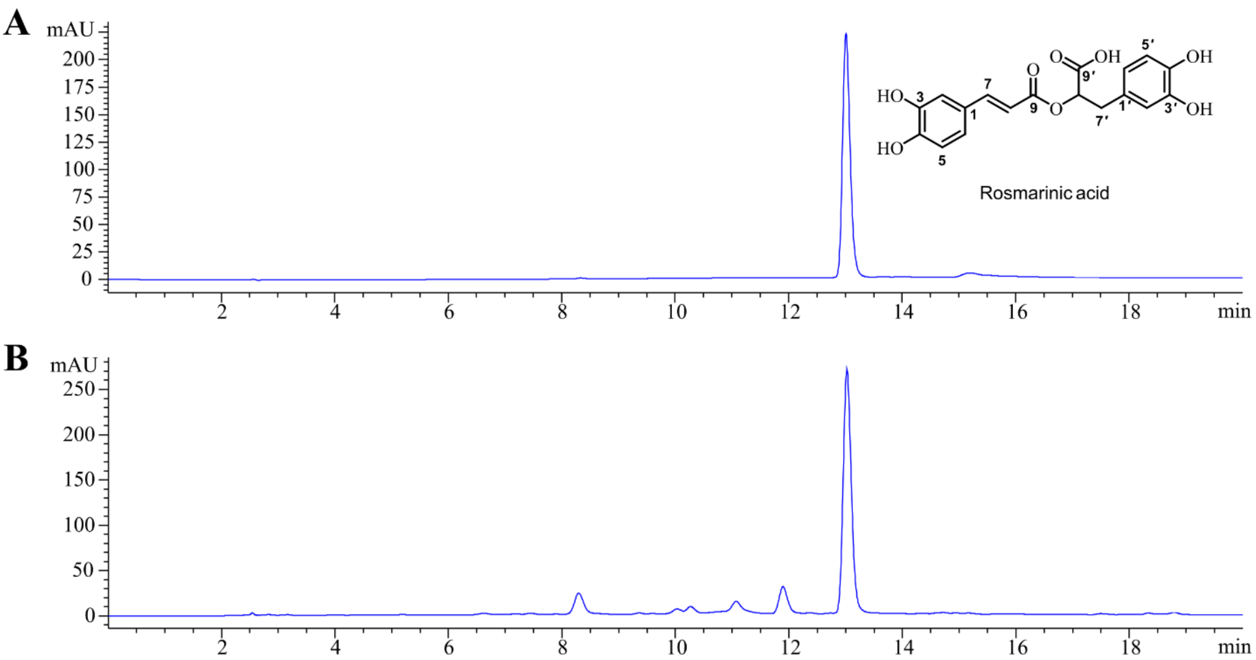

Figure S1 HPLC quantitative analysis of rosmarinic acid (RA) in *T. laurifolia* extract. (**A**) A chromatogram of an RA peak with a retention time (t_R) at 13.0 min. (**B**) A chromatogram of a *T. laurifolia* NADES extract showing an RA peak at the same t_R.

Table S1. Properties of calibration curves, calculated LOD, LOQ, and range of quantitation of rosmarinic acid

Properties	
Regression equation	y = 27.326x - 27.715
Coefficient of determination (R^2)	0.99998
Standard deviation of signal (σ)	33.3099
Limit of detection (LOD)	4.671 µg mL^{-1}
Limit of quantitation (LOQ)	13.20 µg mL^{-1}
Range of quantitation	13.20 – 500 µg/mL

Table S2. Intra-day and inter-day precision of rosmarinic acid**.**

Exp.	Intra-day precision			Inter-day precision		
	Average measured conc. (μg/mL)	SD[a]	%RSD[b]	Average measured conc. (μg/mL)	Mean± SD[a]	%RSD[b]
1	5.21	0.03	0.58	5.28	0.03	0.57
2	9.02	0.14	1.55	9.05	0.12	1.33
3	30.45	0.32	1.05	31.1	0.73	2.35
4	50.77	0.21	0.41	51.82	1.46	2.82
5	99.19	0.29	0.29	101.77	3.74	3.67
6	301.24	1.57	0.52	304.86	2.46	0.81
7	499.32	11.83	2.37	499.99	1.42	0.28

[a]SD: Standard Deviation

[b]%RSD: Relative Standard Deviation

Table S3. Accuracy test result of rosmarinic acid**.**

	Exp.	Sample concentration (μg/mL)	Added concentration (μg/mL)	Measured concentration (μg/mL)	Recovery (%)±SD[a]	%RSD[b]
Rosmarinic acid	1	107.01	25.00	133.25	104.96±1.16	1.10
	2	107.01	125.00	217.44	88.34±1.77	2.01
	3	107.01	250.00	334.69	91.07±0.31	0.34

[a]SD: Standard Deviation

[b]%RSD: Relative Standard Deviation

Table S4. Experimental plan used for the optimization by Box-Behnken Design (BBD).

Run	Water content	Liquid-to-solid ratio	Temperature	Extraction time
1	40	20	60	35
2	40	20	60	25
3	40	20	80	35
4	40	20	80	25
5	40	10	70	35
6	40	10	70	25
7	40	30	70	35
8	40	30	70	25
9	40	10	60	30
10	40	10	80	30
11	40	30	60	30
12	40	30	80	30
13	30	20	70	35
14	30	20	70	25
15	50	20	70	35
16	50	20	70	25
17	30	20	60	30
18	30	20	80	30
19	50	20	60	30
20	50	20	80	30
21	30	10	70	30
22	30	30	70	30
23	50	10	70	30
24	50	30	70	30
25	40	20	70	30
26	40	20	70	30
27	40	20	70	30

Table S5. ANOVA of quadratic model from BBD design for rosmarinic acid content

Source	SumSq	DF	MeanSq	F	pValue
W (water content)	0.068221	1	0.068221	23.271	0.00041621
L (liquid-to-solid ratio)	0.11162	1	0.11162	38.074	4.80E-05
T (temperature)	0.21299	1	0.21299	72.654	1.95E-06
M (extraction time)	0.026183	1	0.026183	8.9313	0.011305
W·L	0.010257	1	0.010257	3.4988	0.085994
W·T	0.0061219	1	0.0061219	2.0882	0.17404
L·T	0.00065643	1	0.00065643	0.22391	0.64457
W·M	0.0087129	1	0.0087129	2.972	0.11036
L·M	0.00014568	1	0.00014568	0.049692	0.82735
T·M	0.0026209	1	0.0026209	0.894	0.36305
W^2	0.13152	1	0.13152	61.919	4.45E-06
L^2	0.01797	1	0.01797	6.1296	0.029181
T^2	0.035416	1	0.035416	12.081	0.0045822
M^2	0.027898	1	0.027898	9.5162	0.0094527
Total	0.66747	26	0.025672		
Model	0.63229	14	0.045163	15.406	1.50E-05
Linear	0.41902	4	0.10475	35.733	1.41E-06
Nonlinear	0.21327	10	0.021327	7.2748	0.0010111
Residual	0.035179	12	0.0029316		
Lack of fit	0.031874	10	0.0031874	1.9289	0.38937
Pure error	0.0033049	2	0.0016524		

Table S6. Estimated Coefficients and statistics of each coefficient for quadratic model from BBD design for rosmarinic acid content

Coefficient of	value	SE	tStat	pVaIue
Intercept	-5.0306	2.4463	-2.0564	0.06216
W (water content)	0.15080	0.0317	4.7531	0.00046963
L (liquid-to-solid ratio)	0.058465	0.0288	2.0286	0.065292
T (temperature)	0.12968	0.0386	3.3593	0.0056819
M (extraction time)	0.25368	0.0721	3.5183	0.0042364
W·L	-0.00050639	0.0003	-1.8705	0.085994
W·T	0.00039121	0.0003	1.4451	0.17404
L·T	-0.0001281	0.0003	-0.47319	0.64457
W·M	-0.00093343	0.0005	-1.724	0.11036
L·M	0.0001207	0.0005	0.22292	0.82735
T·M	-0.00051194	0.0005	-0.94552	0.36305
W^2	-0.0018449	0.0002	-7.8689	4.45E-06
L^2	-0.00058046	0.0002	-2.4758	0.029181
T^2	-0.00081489	0.0002	-3.4757	0.0045822
M^2	-0.002893	0.0009	-3.0848	0.0094527
Number of observations	27			
Error degrees of freedom	12			
Root Mean Squared Error	0.0541			
R-squared	0.947			
Adjusted R-Squared	0.886			
Predicted R-squared	0.714			
F-statistic vs. constant model	15.4			1.5e-05

Table S7. ANOVA of quadratic model from BBD design for DPPH scavenging activity

Source	SumSq	DF	MeanSq	F	pValue
W (water content)	0.0011294	1	0.0011294	0.0032152	0.95572
L (liquid-to-solid ratio)	1219.6	1	1219.6	3472.1	3.77E-16
T (temperature)	1.6511	1	1.6511	4.7004	0.050974
M (extraction time)	0.79486	1	0.79486	2.2629	0.15837
W·L	0.14445	1	0.14445	0.41122	0.53341
W·T	0.20427	1	0.20427	0.58153	0.46044
L·T	0.18612	1	0.13612	0.52987	0.48063
W·M	0.060775	1	0.060775	0.17302	0.68479
L·M	3.1094	1	3.1094	8.852	0.011588
T·M	0.013552	1	0.013552	0.038582	0.84757
W^2	0.05472	1	0.05472	0.15578	0.69999
L^2	31.003	1	31.003	38.262	7.00E-07
T^2	0.27099	1	0.27099	0.77146	0.39701
M^2	2.1419	1	2.1419	6.0976	0.02953
Total	1277.4	26	49.13		
Model	1273.2	14	90.941	258. 9	1.17E-12
Linear	1222	4	305.51	869.76	1. 1513E-14
Nonlinear	51.119	10	5.1119	14.553	3.13E-05
Residual	4.2151	12	0.35126		
Lack of fit	3.8411	10	0.38411	2.0539	0.37161
Pure error	0.37403	2	0.18701		

Table S8. Estimated Coefficients and statistics of each coefficient for quadratic model from BBD design for DPPH scavenging activity

Coefficient of	value	SE	tStat	pValue
Intercept	33.396	26.777	1.2472	0.23613
W (water content)	0.19009	0.34727	0.54737	0.59416
L (liquid-to-solid ratio)	1.3686	0.31547	4.3381	0.00096483
T (temperature)	-0.26616	0.42256	-0.62986	0.54059
M (extraction time)	-1.805	0.78924	-2.287	0.041154
W·L	-0.0019003	0.0029634	-0.64126	0.53341
W·T	-0.0022598	0.0029634	-0.76258	0.46044
L·T	0.0021571	0.0029634	0.72792	0.48063
W·M	-0.0024652	0.0059267	-0.41595	0.68479
L·M	0.017633	0.0059267	2.9752	0.011588
T·M	0.0011641	0.0059267	0.19642	0.84757
W^2	0.0010129	0.0025664	0.39469	0.69999
L^2	-0.02411	0.0025664	-9.3948	7.00E-07
T^2	0.0022541	0.0025664	0.87833	0.39701
M^2	0.025349	0.010265	2.4693	0.02953
Number of observations	27			
Error degrees of freedom	12			
Root Mean Squared Error	0.593			
R-squared	0.997			
Adjusted R-Squared	0.993			
Predicted R-squared	0.929			
F-statistic vs. constant model	259			1.17e-12

Table S9. The price per unit of chemicals used as an ingredient for NADES in this study

			**Price per unit (THB)**		
**Chemical**	**Reference 1**	**Reference 2**	**Reference 3**	**Reference 4**	**Reference 5**
Choline chloride	595	n/a	n/a	n/a	n/a
Propanediol	295	n/a	321	n/a	590
Glycerol	80	79	n/a	80	53
Propylene glycol	145	101	161	115	104
Lactic acid	214	187	188	178	n/a
Glucose	74	53	47 (monohydrated)	59	53
Sorbitol	115 (70% sol.)	82	70 (70% sol.)	74	98 (70% sol.)
Citric acid	165	115	193	120	129
EtOH	164 (per litre)	73 (per litre)	134	n/a	62 (per litre)

[a]The price is represented per kilogram or as stated in parentless.
[b]The chemicals are in a solid, anhydrous form or as stated in parentless.

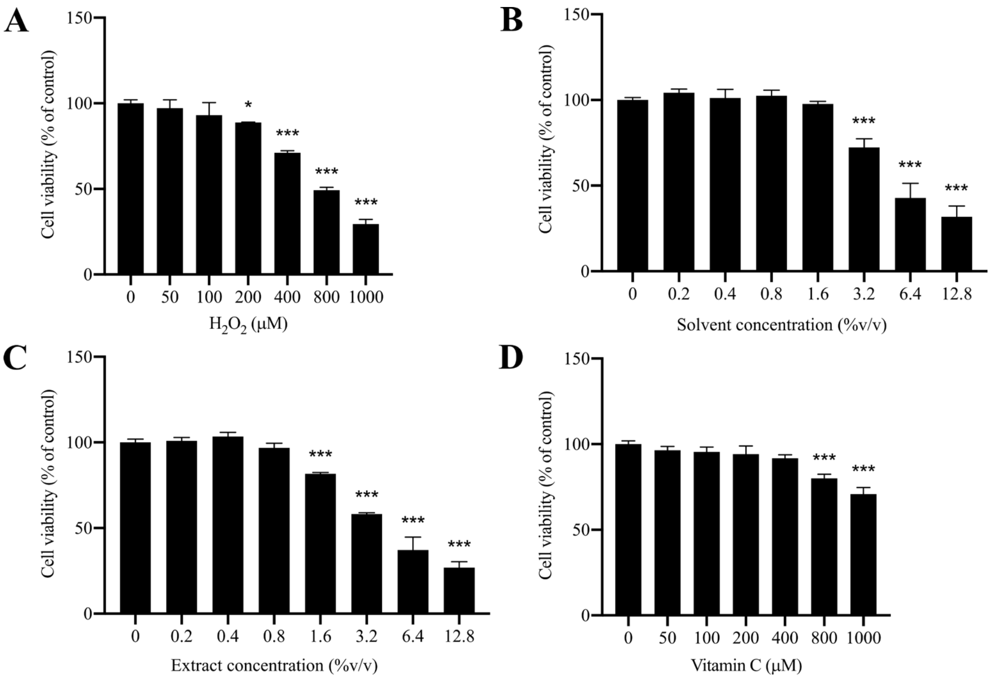

Figure S2. Effect of a 24-hour treatment with test compounds on the viability of keratinocytes. The viability of cells treated with (**A**) H_2O_2, (**B**) NADES solvent, (**C**) plant extract, and (**D**) vitamin C was determined using the MTT assay. All data are expressed as a

percentage relative to the untreated control group. Values are presented as the mean ± SD ($n = 3$). *$p < 0.05$ and ***$p < 0.001$ indicate a significant difference.
